# The Respiratory Pathogen *Moraxella catarrhalis* Targets Collagen for Maximal Adherence to Host Tissues

**DOI:** 10.1128/mBio.00066-16

**Published:** 2016-03-22

**Authors:** Birendra Singh, Maria Alvarado-Kristensson, Martin Johansson, Oskar Hallgren, Gunilla Westergren-Thorsson, Matthias Mörgelin, Kristian Riesbeck

**Affiliations:** a Clinical Microbiology, Department of Translational Medicine, Lund University, Malmö, Sweden; b Molecular Pathology, Department of Translational Medicine, Lund University, Malmö, Sweden; c Respiratory Medicine and Allergology, Department of Experimental Medical Sciences, Lund University, Lund, Sweden; d Section of Infectious Medicine, Department of Clinical Sciences, Lund University, Lund, Sweden

## Abstract

*Moraxella catarrhalis* is a human respiratory pathogen that causes acute otitis media in children and is associated with exacerbations in patients suffering from chronic obstructive pulmonary disease (COPD). The first step in *M. catarrhalis* colonization is adherence to the mucosa, epithelial cells, and extracellular matrix (ECM). The objective of this study was to evaluate the role of *M. catarrhalis* interactions with collagens from various angles. Clinical isolates (*n* = 43) were tested for collagen binding, followed by a detailed analysis of protein-protein interactions using recombinantly expressed proteins. *M. catarrhalis*-dependent interactions with collagen produced by human lung fibroblasts and tracheal tissues were studied by utilizing confocal immunohistochemistry and high-resolution scanning electron microscopy. A mouse smoke-induced chronic obstructive pulmonary disease (COPD) model was used to estimate the adherence of *M. catarrhalis* in vivo. We found that all *M. catarrhalis* clinical isolates tested adhered to fibrillar collagen types I, II, and III and network-forming collagens IV and VI. The trimeric autotransporter adhesins ubiquitous surface protein A2 (UspA2) and UspA2H were identified as major collagen-binding receptors. *M. catarrhalis* wild type adhered to human tracheal tissue and collagen-producing lung fibroblasts, whereas UspA2 and UspA2H deletion mutants did not. Moreover, in the COPD mouse model, bacteria devoid of UspA2 and UspA2H had a reduced level of adherence to the respiratory tract compared to the adherence of wild-type bacteria. Our data therefore suggest that the *M. catarrhalis* UspA2 and UspA2H-dependent interaction with collagens is highly critical for adherence in the host and, furthermore, may play an important role in the establishment of disease.

## IMPORTANCE

The respiratory tract pathogen *Moraxella catarrhalis* adheres to the host by interacting with several components, including the ECM. Collagen accounts for 30% of total body proteins, and therefore, bacterial adherence to abundant host collagens mediates bacterial persistence and colonization. In this study, we characterized previously unknown *M. catarrhalis*-dependent interactions with host collagens and found that the trimeric autotransporter adhesins ubiquitous surface protein A2 (UspA2) and UspA2H are highly important. Our observations also suggested that collagen-mediated adherence of *M. catarrhalis* is indispensable for bacterial survival in the host, as exemplified by a mouse COPD model.

## INTRODUCTION

*Moraxella catarrhalis* is a Gram-negative diplococcus that colonizes the human respiratory tract. The pathogen causes acute otitis media in children and is also associated with bronchitis, sinusitis, laryngitis, and exacerbations in patients with chronic obstructive pulmonary disease (COPD) (1–4). It is frequently found in coinfections with *Haemophilus influenzae* and/or *Streptococcus pneumoniae.*

The first step of a successful colonization is adhesion to the host mucosal surface, epithelial cells, and finally, extracellular matrix (ECM). Adhesion is mediated by an array of outer membrane proteins (3), including the *M. catarrhalis* ubiquitous surface proteins (Usp). This protein family comprises well-characterized trimeric autotransporter adhesins (TAAs), which are recognized as multifunctional virulence factors of *M. catarrhalis* (5–9). Ubiquitous surface proteins occur as lollipoplike structures that consist of a membrane anchor, stalk, neck, and head domain on the outer membrane (10). They are further divided into three subgroups, as follows: (i) ubiquitous surface protein A1 (UspA1) is present in all clinical isolates; (ii) UspA2 is found in 75% of strains; and finally, (iii) approximately 25% of clinical isolates carry UspA2H instead of UspA2. Ubiquitous surface protein A1 binds to CAECAM-1 surface receptors, and hence, plays an important role in bacterial colonization (11). Either UspA2 or both UspA1 and UspA2 interact with components of the complement pathway to protect *M. catarrhalis* from the bactericidal activity of serum, and they also bind to ECM components to enhance adherence to the host (5–8, 12–15).

Collagens are the most abundant glycoproteins of the human body and account for 30% of the total protein mass involved in the formation of structural scaffolds, cell adhesion, and angiogenesis and the development of organs (16, 17). The basic structural unit of a collagen molecule consists of an α-chain (monomer). The α-chains associate into a trimer in a triple-helix form to build a protomer, and these associate further, forming a supramolecular organization. On the basis of their supramolecular arrangements, collagens have been categorized into the following five different groups: fibril-forming collagens, network-forming collagens, FACITs (fibril-associated collagen with interrupted triple helices), MACITs (membrane-associated collagen with interrupted triple helices), and multiplexins (16). The triple-helix region of the α-chains consists of the recurring triplet Gly-X-Y repeated *n* times in the central part of each α-chain, with X and Y often being proline and hydroxyproline, respectively. The other domains of α-chains, which have ordinary amino acid sequences, are termed noncollagenous (NC) domains and are present at the N- or C-terminal end of α-chains. The NC domains are cleaved off in some protomers (fibril-forming collagens), while they are retained in the network-forming collagens (17).

An individual collagen does not represent a tissue’s specificity; rather, several types of collagens associate to form complex structures by defined protein-protein interactions (17–19). Type I collagen is one of the most abundant, present in all human tissues, whereas type II collagen forms fibrils and associates with hyaline and elastic cartilages, including tracheal cartilage (20). Type III collagen is present in connective tissues (reticulate fibers), such as the skin, lungs, and the vascular system (21). The epithelium of all organs anchors to the underlying tissues by interacting with the basement membrane (BM), which is composed of laminin and collagen IV networks (22). Collagen type VI is abundantly present in the ECM of the respiratory system and is upregulated in COPD (23).

It has previously been shown that *M. catarrhalis* adheres to collagen IV by its outer membrane IgD-binding protein (MID/Hag) (24). Here, we studied in detail the interactions of *M. catarrhalis* with various human collagens and further investigated their *in vivo* relevance. The abundant collagens, including fibril-forming types I, II, and III and network-forming types IV, V, VI, and VIII, were tested for binding with several clinical isolates. Bacterial surface proteins interacting with collagens were identified, and protein-protein interactions were elucidated at the molecular level. Moreover, the biological significance of *M. catarrhalis*-dependent collagen interactions was verified by using human fibroblasts and upper respiratory tract tissue and was finally examined in an *in vivo* COPD mouse model. Our results indicate that *M. catarrhalis* efficiently targets collagens for optimal adherence to host tissues.

## RESULTS

### *M. catarrhalis* binds to different fibril- and network-forming collagens.

**FIG 1:**
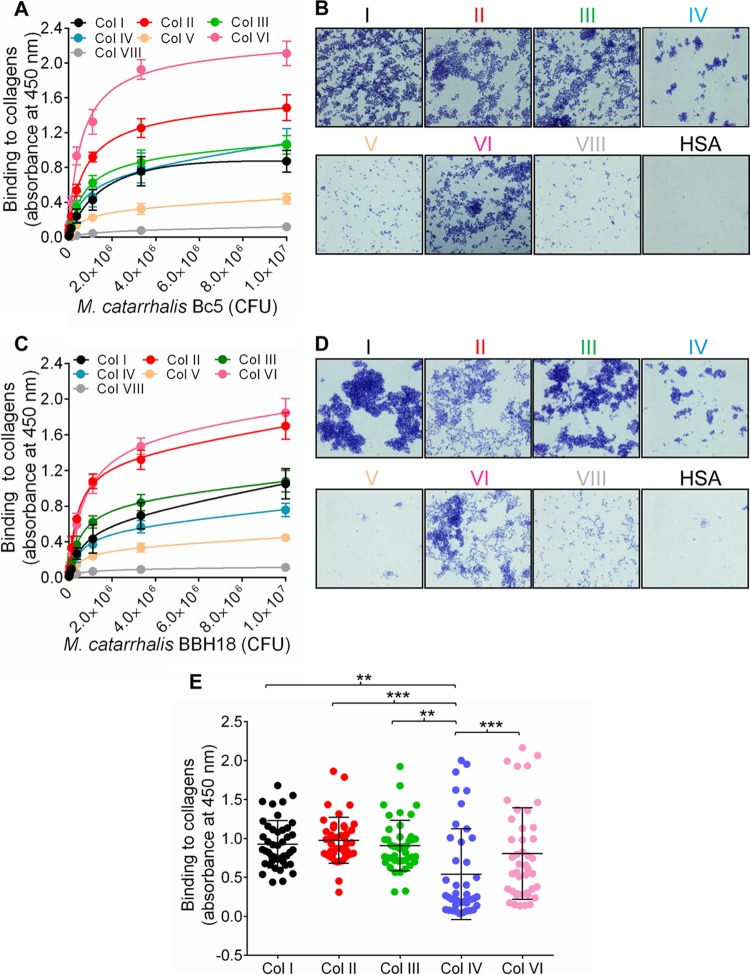
*Moraxella catarrhalis* adheres to various human collagens. (A) Dose-dependent binding of *M. catarrhalis* Bc5 to purified collagens (50 nM) as revealed by ELISA. ELISA plates were coated with collagens I, II, III, IV, VI, and VIII, and serial dilutions of *M. catarrhalis* Bc5 were added. Bound bacteria were detected by anti-*Moraxella* PAbs. (B) Adherence of *M. catarrhalis* Bc5 to collagen-coated glass slides. Glass slides were coated with 2 µg collagen or HSA, followed by incubation with 10^7^ CFU/ml bacteria in BHI medium. Bacteria were visualized by Gram staining and light microscopy. (C) Binding of *M. catarrhalis* BBH18 to purified collagens I, II, III, IV, V, VI, and VIII as analyzed by ELISA. (D) Adherence of *M. catarrhalis* BBH18 to collagen-coated glass slides. (A and C) Data shown are the means of the results from three independent experiments, and error bars represent standard deviations. (A and B) *M. catarrhalis* bacteria (3.0 × 10^5^ to 1.0 × 10^7^ CFU added) bound to collagens I, II, III, IV, and VI significantly more than to collagens V and VIII (*P* value range, 0.05 to 0.001; two-way ANOVA). (E) *Moraxella catarrhalis* clinical isolates bind to human collagens. The adherence of *M. catarrhalis* isolates (10^6^ CFU) to various collagens (50 nM) was analyzed by ELISA. The means of the results of three independent experiments performed in triplicates are shown and error bars indicate standard deviations. Multiple comparisons were done with one-way ANOVA. *, *P* ≤ 0.05; **, *P* ≤ 0.01; ***, *P* ≤ 0.001.

*M. catarrhalis* Bc5 and BBH18 wild-type (WT) strains were chosen to test interactions with various collagens. Increasing concentrations of bacteria were added to microtiter plates that had been precoated with collagens. Unbound bacteria were removed by washing, and attached bacteria were analyzed by using anti-*Moraxella* polyclonal antibodies (PAbs). Our results revealed significant adherence of *M. catarrhalis* Bc5 to most collagens tested. However, *M. catarrhalis* Bc5 bound more to collagens II and VI than to collagens I, III, and IV, whereas collagens V and VIII were not targeted by *M. catarrhalis* (Fig. 1A). In parallel with the enzyme-linked immunosorbent assay (ELISA), we coated glass slides with collagen and visualized the adherence of *M. catarrhalis* by Gram staining and microscopy. Similar results were observed using this method (Fig. 1B). The other *M. catarrhalis* strain, BBH18, also adhered to the collagens at significant levels (Fig. 1C and D). In contrast, *M. catarrhalis* did not adhere to human serum albumin (HSA), which was included as a negative control (Fig. 1B and D). These results clearly indicated that *M. catarrhalis* has a selective specificity for fibrillar collagens I, II, and III, in addition to network-forming collagens IV and VI. Since the remaining network-forming collagens, types V and VIII, were not targeted by *M. catarrhalis*, we excluded those from further downstream analyses.

In the next set of experiments, various clinical isolates of *M. catarrhalis* (5, 25) were selected to verify interactions with collagens. All *M. catarrhalis* clinical isolates tested bound collagens I, II, III, IV, and VI, but the binding capacity varied between isolates. The mean levels of binding of *M. catarrhalis* to collagens I, II, III, and VI were significantly higher than the mean level of binding to collagen IV (Fig. 1E). A comparison of various clinical isolates regarding their collagen-binding capacities is shown in Fig. S1 in the supplemental material. Our data thus indicated that clinical *M. catarrhalis* isolates recognize several human collagens that may contribute to bacterial adherence.

### The trimeric autotransporters UspA2 and UspA2H are major collagen-binding proteins of *M. catarrhalis.*

**FIG 2:**
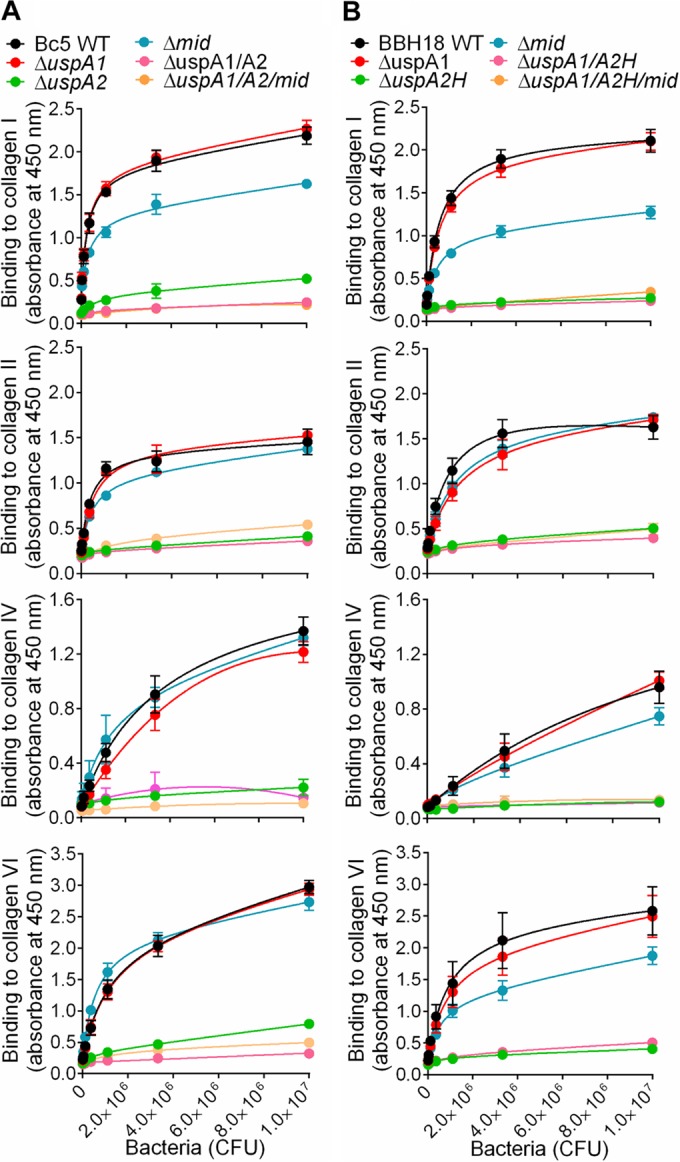
UspA2 and UspA2H are collagen-binding proteins of *M. catarrhalis*. (A) Results from ELISA showing adherence of *M. catarrhalis* Bc5 and isogenic single and multiple mutants to collagens. (B) Adherence of *M. catarrhalis* BBH18 and isogenic single and multiple mutants to collagens. The means of the results of three independent experiments are shown here, and error bars represent standard deviations. Multiple comparisons were done by a two-way ANOVA. *M. catarrhalis* (3.0 × 10^5^ to 1.0 × 10^7^ CFU added) Bc5 Δ*uspA2*, Δ*uspA1* Δ*uspA2*, and Δ*uspA1* Δ*uspA2* Δ*mid* mutants had significantly reduced binding to collagens in comparison to the binding of Bc5 WT and the Δ*uspA1* and Δ*mid* mutants (*P* value range, 0.05 to 0.001). In parallel, *M. catarrhalis* BBH18 Δ*uspA2H*, Δ*uspA1* Δ*uspA2H*, and Δ*uspA1* Δ*uspA2H* Δ*mid* mutants bound significantly less to collagens than did BBH18 WT and Δ*uspA1* and Δ*mid* mutants (*P* value range, 0.05 to 0.001).

Trimeric autotransporter adhesins, as exemplified by *Yersinia enterocolitica* YadA (39), *Aggregatibacter actinomycetemcomitans* EmaA (26), and *Bartonella hensalae* BadA (27), bind human collagens. Similarly, the high-molecular-weight TAA MID of *M. catarrhalis* interacts with collagen IV (24). These previous studies prompted us to investigate whether *M. catarrhalis* TAAs also recognize an array of human collagen molecules. We performed an ELISA to screen single and multiple mutants of *M. catarrhalis* TAAs. Interestingly, selective deletion of UspA2 in *M. catarrhalis* Bc5 (Δ*uspA2*) resulted in significantly reduced binding to collagens I, II, IV, and VI (Fig. 2A). In contrast to the UspA2 mutant (*M. catarrhalis* Bc5 Δ*uspA2*), the mutant devoid of MID (Δ*mid*) had slightly decreased binding to collagen I but not to the other collagens tested (Fig. 2A). Similar results were obtained with *M. catarrhalis* BBH18 WT and the Δ*uspA2* and Δ*uspA2H* mutants (Fig. 2B). Finally, mutants with deletion of UspA2 or UspA2H in combination with deletion of UspA1 and MID were analyzed, proving that UspA2 and UspA2H played the largest role in *Moraxella*-dependent adherence to collagens (Fig. 2).

**FIG 3:**
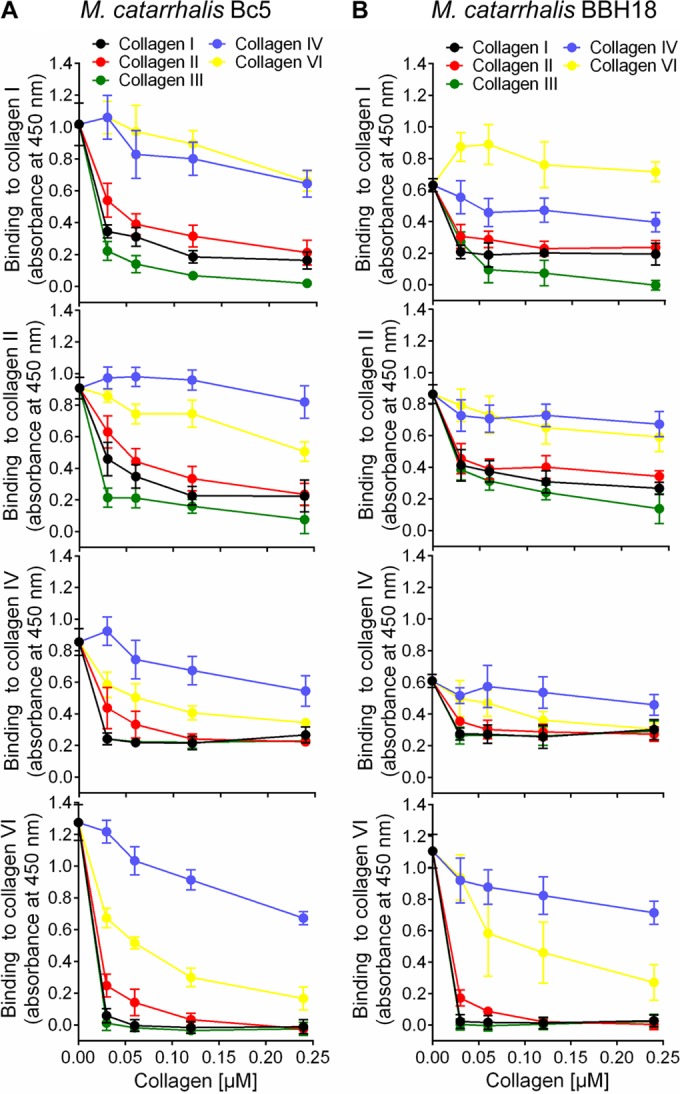
UspA2 and UspA2H have the same binding sites for fibrillar collagens (I, II, and III) and network-forming collagens (IV and VI). (A) Inhibition of *M. catarrhalis* Bc5 adhesion to immobilized collagens I, II, IV, and VI by the soluble collagens indicated in the key. (B) Inhibition of *M. catarrhalis* BBH18 adhesion to immobilized collagens I, II, IV, and VI by various soluble collagens. In A and B, the indicated collagens were coated to microtiter plates followed by ELISA.

To verify the specificity of UspA2- and UspA2H-dependent collagen binding, we performed a competitive inhibition assay. Fibrillar collagens I, II, and III significantly inhibited the adherence of *M. catarrhalis* Bc5 and BBH18 to collagens I, II, IV, and VI in comparison to the inhibition by the network-forming collagens IV and VI (Fig. 3A and B). Moreover, fibrillar collagens also blocked the interaction of network-forming collagens (Fig. 3A and B), confirming that both UspA2 and UspA2H have a single domain interacting with collagen. Taken together, our results revealed that UspA2 and UspA2H are the major collagen-binding proteins in *M. catarrhalis* and that fibrillar collagens are prime targets for optimal adherence.

### *M. catarrhalis* UspA2 and UspA2H target gap regions of fibrillar collagens.

**FIG 4:**
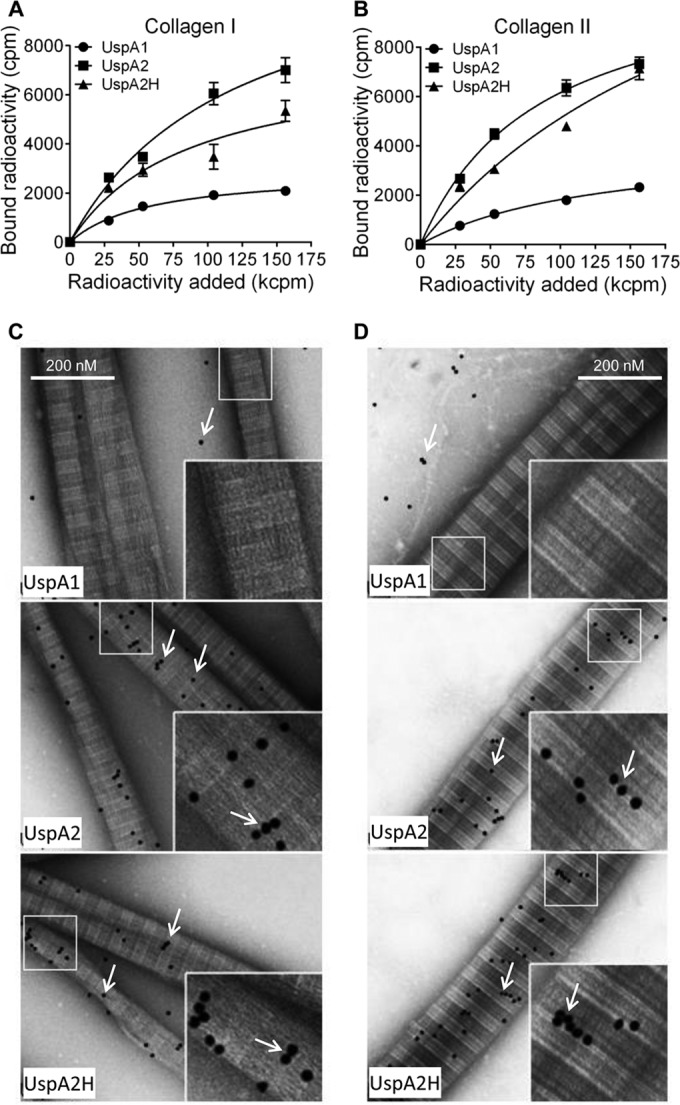
Recombinant *M. catarrhalis* UspA2 and UspA2H bind to fibrillar collagens I and II. (A and B) Binding of recombinant ^125^I-labeled UspA1, UspA2, and UspA2H to collagen I (A) or collagen II (B). ^125^I-labeled UspA2 and UspA2H (28 to 156 kcpm added) showed significantly higher levels of binding to collagens I and II than did UspA1 (*P* value range, 0.01 to 0.001). Mean values from three independent experiments in triplicates are shown. Error bars indicate standard deviations. Multiple comparisons were performed using two-way ANOVA. (C and D) TEM images showing binding of gold-labeled UspA1, UspA2, and UspA2H to a grid coated with collagen I (C) or collagen II (D). The gold particles, appearing as black dots and indicated by arrows, show UspA2 and UspA2H bound to collagen fibrils.

To define molecular interactions between UspA2 and UspA2H and the different collagens, we performed a series of protein-protein interaction studies using a direct binding assay. ^125^I-labeled recombinant UspA2, UspA2H, and UspA1 were added to microtiter plates coated with collagen. The unbound fractions were removed by washing, and bound proteins were estimated by scintillation counting. In contrast to the results for UspA1, which was used as a negative control, a dose-dependent binding of UspA2 and UspA2H to collagens I and II was observed (Fig. 4A and B).

Collagens are large molecules that can be easily visualized in transmission electron microscopy (TEM). The triple helices of collagens I and II overlap to provide variable thickness of the fibril. In TEM analysis, a light-gray short span between light strands represents the overlapping region, whereas the dark-gray span represents the gap regions (18). Ubiquitous surface proteins were labeled with gold particles (10 nm), added to grids with collagens I or II, and visualized by negative staining. Interestingly, UspA2 and UspA2H bound directly to fibrillar collagens I and II at overlap regions of the triple helices (Fig. 4C and D). Quantification of gold particles in 50 randomly selected regions revealed significantly higher levels of binding of UspA2 and UspA2H than of UspA1 to collagens I and II (see Fig. S2A in the supplemental material). These data were in agreement with the results of our direct binding assay (Fig. 4A and B).

### UspA2 and UspA2H bind to NC domains of network-forming collagens IV and VI.

**FIG 5:**
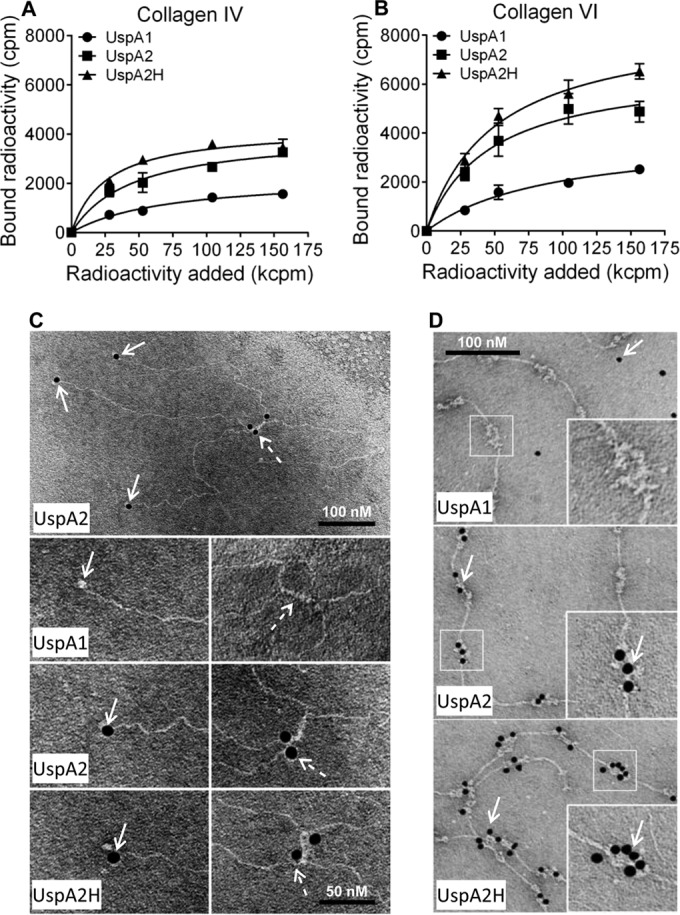
Recombinant UspA2 and UspA2H bind to network-forming collagens IV and VI. (A and B) Binding of recombinant ^125^I-labeled UspA1, UspA2 and UspA2H to collagen IV (A) or collagen VI (B) coated in microtiter plates. The means of the results of three independent experiments in triplicates are shown. The error bars indicate standard deviations. Statistical calculations were performed using two-way ANOVA. The differences between ^125^I-labeled UspA2 and UspA2H and UspA1 (28 to 156 kcpm added) in binding to collagen IV and VI were statistically significant (*P* value range, 0.01 to 0.001). (C) TEM images of gold-labeled UspA1, UspA2, and UspA2H binding to a collagen IV-coated grid. The solid arrows indicate gold particles bound to NC domains, and dashed arrows show N7 domains of the collagen IV molecule. (D) Binding of gold-labeled UspA1, UspA2, and UspA2H to a collagen VI-coated grid as revealed by TEM. Gold particles are indicated by arrows.

We also measured the interaction of UspAs with network-forming collagens IV and VI by a direct binding assay. ^125^I-labeled UspA2 and UspA2H bound to these collagens in a dose-dependent manner (Fig. 5A and B). In parallel with the results for fibrillar collagens, UspA1 did not bind collagens IV and VI. Another observation was that *M. catarrhalis* UspA2 and UspA2H bound slightly more to collagen VI than to collagen IV, a finding that correlated with our findings with intact bacteria (Fig. 2).

The collagen type IV network starts with the formation of dimers of two protomers at their NC domains. In a second phase, dimers interact through their 7S domain (N-terminal NC plus the N-terminal part of the triple helix) to form tetramers (18). UspA2 and UspA2H interacted with the NC domain (dotted arrows) and the N-terminal part (solid arrows) of tetramers (Fig. 5C). The NC and 7S domains, which are nonhelical structures, were thus targeted by *M. catarrhalis*.

Collagen VI consists of a filamentlike structure with N-terminal and C-terminal noncollagenous domains designated von Willebrand (WVA) domains. It is arranged into tetramers by a combination of four α-chains. The tetramers further polymerize to constitute a beaded filamentlike structure (23). Interestingly, gold-labeled UspA2 and UspA2H only localized to the noncollagenous WVA domains (Fig. 5D). The helical collagenous region was not targeted by either UspA2 or UspA2H. When gold granules were quantified, a significantly higher number of UspA2 and UspA2H molecules than of UspA1 molecules were found to have bound to collagens IV and VI (see Fig. S2B in the supplemental material). This observation supported our results obtained by direct binding of iodine-labeled proteins (Fig. 5A and B). In conclusion, our results indicated that UspA2 and UspA2H specifically bind to the noncollagenous domains of collagens IV and VI.

### *M. catarrhalis* adheres to collagens in human lung fibroblasts and trachea.

**FIG 6:**
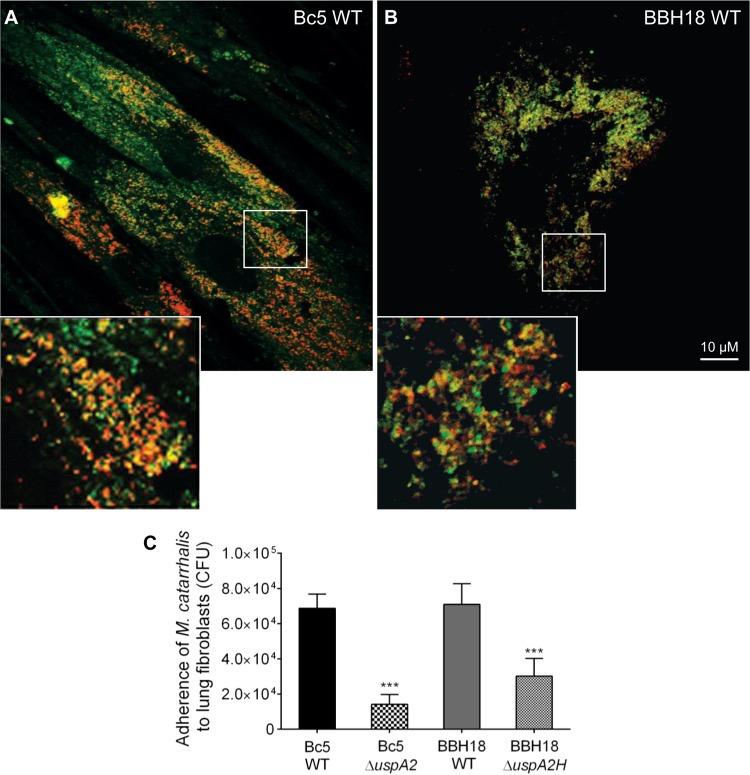
Adherence of *M. catarrhalis* bacteria to primary fibroblasts is dependent on UspA2 and UspA2H, as revealed by confocal microscopy. (A and B) Adherence of *M. catarrhalis* Bc5 WT (A) and BBH18 WT (B) to fibroblasts. A detailed outline of the experiment, in addition to the results for *M. catarrhalis* Δ*uspA2* and Δ*uspA2H* mutants, is presented in Fig. S3 in the supplemental material. Fibroblasts were incubated with FM 4-64-labeled bacteria (red). After washing, the ECM, including collagen fibrils, were visualized by FITC-labeled UspA2^30–539^ (green). Overlays resulting in orange color illustrate colocalization of *M. catarrhalis* and collagens, including other components of the ECM. The size bar represents 10 µm. (C) Adherence of *M. catarrhalis* bacteria to fibroblasts as determined by CFU. Data shown are the means of the results of three independent experiments done in triplicates, and error bars represent standard deviations. Statistical analyses were performed using one-way ANOVA. ***, *P* ≤ 0.001.

Since *M. catarrhalis* binds purified collagens *in vitro* (Fig. 1), we wanted also to verify our findings in a fibroblast model. Fibroblasts are found in the lamina propria and produce ECM proteins, including collagens (28). We isolated and expanded primary fibroblasts from lung biopsy specimens and added *M. catarrhalis* cells that had been labeled with Fm4-64 (red dye) (23). Collagen fibrils, including other ECM proteins, were costained with UspA2-fluorescein isothiocyanate (FITC; green dye) and subjected to confocal microscopy. More pronounced adhesion to fibroblasts was observed with *M. catarrhalis* strains Bc5 WT and RH4 WT than with the UspA2 and UspA2H mutants (Fig. 6A and B; see also Fig. S3 in the supplemental material). Importantly, at higher magnifications, we observed that *M. catarrhalis* cells were colocalized with ECM of fibroblasts. These results were also verified by a quantitative adherence assay based upon CFU counting. *M. catarrhalis* Bc5 and BBH18 adhered significantly more than the corresponding mutants (Fig. 6C). Our experiments thus suggested that UspA2 and UspA2H are essential proteins for maximal adherence to lung fibroblasts.

**FIG 7:**
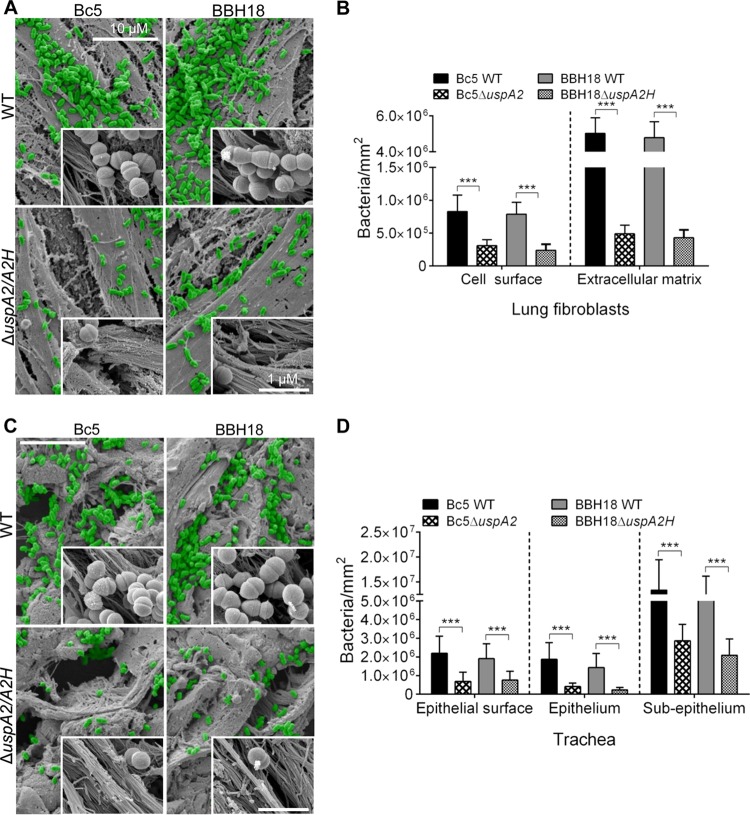
*M. catarrhalis* UspA2- and UspA2H-dependent adhesion to collagens in human lung fibroblasts and trachea. (A) Lung fibroblasts targeted by *M. catarrhalis* WT and UspA2 and UspA2H mutants were analyzed by SEM. Bacteria are visualized in green pseudocolor. (B) Adherence of *M. catarrhalis* strains per mm^2^ of fibroblasts. (C) *M. catarrhalis* WT and UspA2 and UspA2H mutants adhere to human tracheal tissues. (D) Adherence of *M. catarrhalis* strains per mm^2^ of trachea. Error bars indicate standard deviations. Statistical analyses were performed using Student’s *t* test. ***, *P* ≤ 0.001.

To further explore the capacity of *M. catarrhalis* to adhere to fibroblasts, preparations were subjected to SEM. *M. catarrhalis* Bc5 WT and BBH18 WT adhered to the surface of fibroblasts more efficiently than the corresponding UspA2 and UspA2H mutants (Fig. 7A). Several bacteria were easily seen to be colocalized around collagen fibers (Fig. 7A, zoomed insets). Interestingly, *M. catarrhalis* WT strains had approximately 6-fold better adherence to the ECM region than to the surface of the fibroblasts (Fig. 7B).

To estimate the levels of adherence of *M. catarrhalis* strains to primary tissue, sections of human trachea were studied. *M. catarrhalis* strains Bc5 and BBH18 and their corresponding UspA2 and UspA2H mutants were added to tracheal specimens, and the overall bacterial adherence was estimated. *M. catarrhalis* mutants devoid of UspA2 or UspA2H adhered less than the WT bacteria (Fig. 7C). Intriguingly, higher magnification of tissue sections revealed that *M. catarrhalis* adhered to collagen bundles (Fig. 7C, insets). Bacterial adherence was quantified at the surface, epithelial, and subepithelial regions. At the epithelial surface, 3.0-fold more *M. catarrhalis* Bc5 WT was found than for the UspA2 mutant, and in the case of BBH18, 2.5-fold more WT bacteria were observed than for the corresponding UspA2H mutant (Fig. 7D). Similarly, *M. catarrhalis* Bc5 and BBH18 WT bound more to the epithelium than the corresponding UspA2 and UspA2H mutants. Interestingly, *M. catarrhalis* Bc5 and BBH18 adhered to the subepithelial region (lamina propria) approximately 6- to 8-fold more than to the epithelial surface and epithelium (Fig. 7D), an observation that most likely was due to abundant ECM proteins, including collagen. Taken together, our results suggest that *M. catarrhalis* targets host collagens and that the interaction is mediated by a TAA, either UspA2 or UspA2H.

### UspA2 and UspA2H contribute to bacterial adherence and survival in a COPD mouse model.

**FIG 8:**
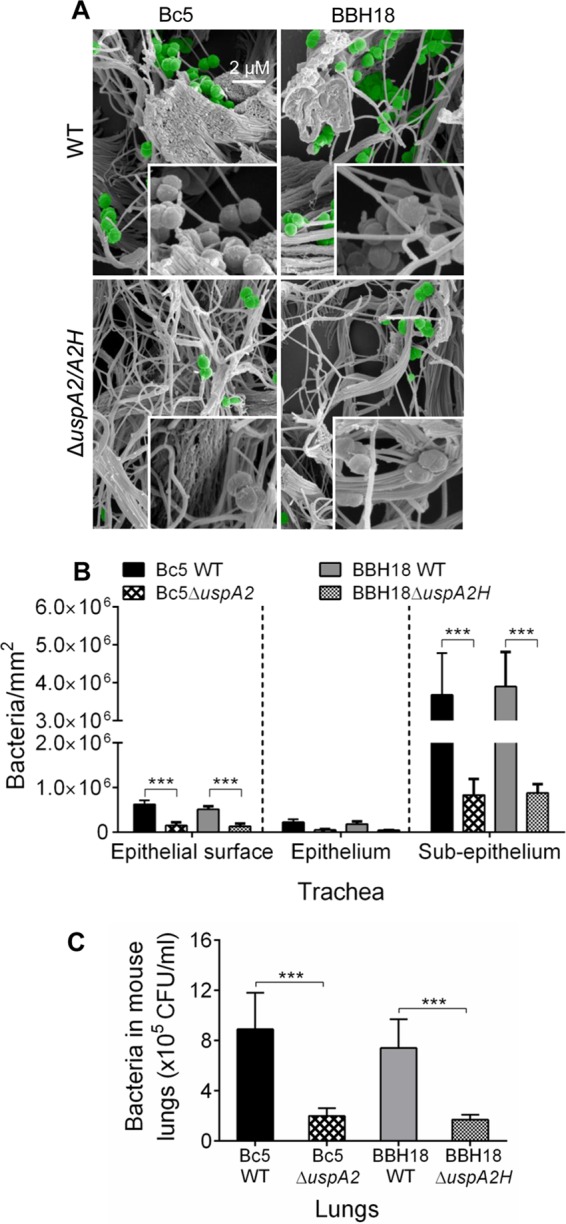
Increased adherence of *M. catarrhalis* to mouse COPD lungs. (A) COPD mice were challenged by intratracheal inoculation of *M. catarrhalis* Bc5 WT and BBH18 WT and the corresponding UspA mutants. After 30 min, tracheal sections were analyzed by SEM. Bacteria are highlighted in green pseudocolor. (B) Quantitative estimation was performed by counting bacterial cells/mm^2^ from randomly selected regions comprising epithelial surface, epithelium, and subepithelium. (C) Excised lungs were homogenized, and bacterial counts were determined by serial dilution and plating. Error bars represent standard deviations. Statistical analyses were performed using Student’s *t* test. ***, *P* ≤ 0.001.

To investigate the *in vivo* relevance of our findings, we challenged mice with smoke-induced COPD with *M. catarrhalis* Bc5 and BBH18 WT strains and their corresponding UspA2 and UspA2H mutants. After 30 min, tracheas were excised and embedded in paraffin, followed by imaging of tissue sections using high-resolution scanning electron microscopy (SEM) (Fig. 8A). As expected, *M. catarrhalis* UspA2 and UspA2H mutants adhered significantly less than the WT bacteria. Bacteria were seen around the collagen fibrils, and adherence was quantified by counting in several randomly selected parts of the tissue sections (Fig. 8B). In parallel with the experiments done *in vitro* (Fig. 6 and 7), we found that most *M. catarrhalis* cells bound to the subepithelial region, which is rich in collagens and other ECM proteins. After tissue homogenization and plating, CFU were counted and revealed higher levels of adherence of UspA2- and UspA2H-expressing *M. catarrhalis* bacteria (Fig. 8C). In summary, our results with mice having a COPD phenotype suggested that upon host colonization, *M. catarrhalis* bacteria most likely rely upon UspA2 and UspA2H for maximal interactions with tissue collagens.

## DISCUSSION

**FIG 9:**
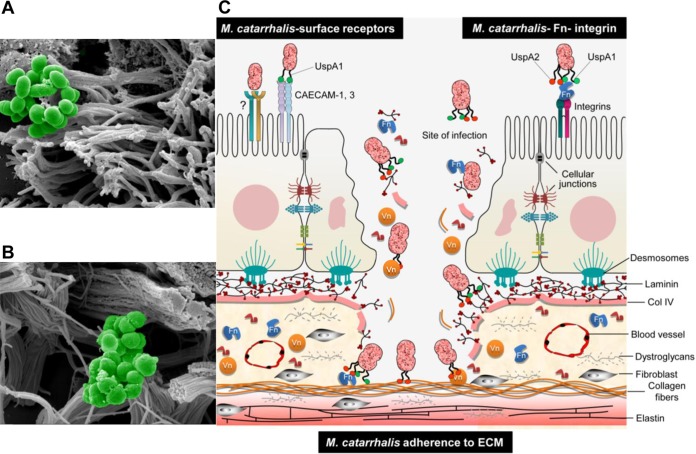
Adherence of *M. catarrhalis* to human tracheal tissue. (A) SEM of section of human trachea incubated with *M. catarrhalis* Bc5 WT and embedded in paraffin. Magnified view of the epithelial surface shows adherence of bacteria to cilia. (B) SEM showing adherence of *M. catarrhalis* Bc5 WT to lamina propria. Collagen bundles are targeted by the bacteria. (C) Cartoon depicting a model of how *M. catarrhalis* adheres and colonizes the human host by using outer membrane proteins for targeting the ECM.

*M. catarrhalis* is mostly associated with infections in children and, occasionally, in adults (1–3). Under various pathogenetic conditions, including COPD and emphysema, tissue fibrosis, where collagen expression increases severalfold, is a frequent observation (29–32). Enhanced levels of collagen would be favorable for increased adherence of *M. catarrhalis*. This bacterial species also adheres to the upper airways by utilizing host surface receptors, such as CAECAM1 to -3 and integrins (via Fn-mediated adherence), which results in a proinflammatory response and, to some extent, bacterial internalization (3). While UspA1 is involved in interactions with epithelial surface receptors, UspA2 and UspA2H recognize predominantly ECM proteins. Our data suggest that *M. catarrhalis* adheres to cilia at the epithelial cell surface (Fig. 9A). In addition to COPD and lung fibrosis, other primary infections of viral origin may also compromise the epithelial cell barrier, which may lead to increased exposure of the lamina propria. In such a scenario, *M. catarrhalis* has the opportunity to attach to ECM proteins, including collagens, laminin, and fibronectin, resulting in increased bacterial persistence (Fig. 9B). In order to visualize our hypothesis, a summarizing cartoon is shown in Fig. 9C. This hypothesis is in part supported by our *in vivo* observations, where COPD model mice with damaged mucosas and changed barriers due to tobacco smoke revealed significantly increased levels of adherence of the bacteria investigated.

It has previously been shown that *M. catarrhalis* can adhere to collagen IV and that this interaction is mediated by the trimeric autotransporter MID (24). Our detailed adherence assay with several *M. catarrhalis* clinical isolates revealed strong binding to fibrillar collagens, while network-forming collagen IV was least recognized. The MID-deficient mutants Bc5 and BBH18 did not show any significant differences in interactions with collagen IV in comparison to the interactions of their wild-type counterparts. As reported by Bullard et al. (24), one *M. catarrhalis* strain, O35E, bound collagen IV via MID. We also found that a few isolates had higher affinities for collagen IV (Fig. 1E). Binding to collagen IV could therefore be related to a few isolates that may reflect various levels of expression of mainly MID. It is evident, however, that *M. catarrhalis* MID and UspAs have varying amino acid sequences with defined motifs (24, 33) and that the expression of UspA is also variable among clinical isolates (25). Moreover, these characteristics of UspA may explain the differences in collagen binding of clinical isolates. According to our results, collagen IV, which is present in the basement membrane, is not a prime target for all clinical isolates.

Among the TAAs, YadA of *Yersinia* has been extensively studied for its interactions with fibrillar collagens and peptides derived from these molecules (34). The head region of YadA binds promiscuously to fibrillar collagen. It easily recognizes the Gly-Pro-Hyp single triplet, and a peptide comprising (Gly-Pro-Hyp)_6_ had a strong interaction with YadA. In fact, the triple-helix regions of fibrillar collagens contain several repeats of Gly-Pro-Hyp, and thus, YadA has been shown to interact at multiple places in the triple helix. In addition, microbial surface components recognizing adhesive matrix molecules (MSCRAMMs) of Gram-positive bacteria, such as Cna from *Staphylococcus aureus*, bind to collagen triple-helix regions (35). UspA2 and UspA2H are structurally diverse molecules regarding domain length and sequence (25, 36). This is the first report demonstrating interactions of *M. catarrhalis* UspA2 and UspA2H with fibrillar and network-forming collagens at the structural level. Based upon our EM data, UspA2 and UspA2H both interacted with collagens I and II at the overlap regions of triple helices, confirming that these interactions indeed took place in the triple-helix region (Fig. 4C and D). In contrast, triple-helix regions of collagens IV and VI are not recognized by UspA2 and UspA2H but, rather, by NC domains of the molecules involved (Fig. 5C and D). It will be intriguing to study the precise domains of collagens or Usp molecules that are involved in the interactions. Moreover, the mechanism of binding between Usp and collagen molecules will be of great interest.

In conclusion, we found that *M. catarrhalis* clinical isolates have a large capacity to adhere to host collagen molecules. The surface TAAs UspA2 and UspA2H are versatile proteins that recognize collagens and promote bacterial adherence to host tissues, consequently increasing survival in the respiratory tract. Our findings on *M. catarrhalis*-dependent interactions with collagens may be highly valuable for the design of future therapies, including vaccines aiming to control this common respiratory pathogen.

## MATERIALS AND METHODS

### Bacterial strains and culture conditions.

*M. catarrhalis* Bc5 and BBH18 and other *Moraxella* clinical isolates (8) were cultured on chocolate agar plates or in brain heart infusion (BHI), followed by incubation at 37°C in a humid atmosphere at 5% CO_2_. The *M. catarrhalis* mutants with single and multiple mutations of *uspA1*, *uspA2*, *uspA2H*, and *mid* were obtained as described elsewhere. According to the presence of resistance markers, the *M. catarrhalis* mutants were grown in 1.5 µg ml^−1^ chloramphenicol, 7 µg ml^−1^ zeocin, or 20 µg ml^−1^ kanamycin. The multiple mutants were grown with combinations of antibiotics. *Escherichia coli* BL21(DE3) was cultured in Luria-Bertani (LB) broth or on LB agar plates at 37°C. *E. coli* bacteria containing pET26b expression vectors with *uspA2* and *uspA2H* genes (6) were grown in LB medium supplemented with 50 µg ml^−1^ kanamycin.

### Collagen adherence assays.

Different purified collagens (types I, II, III, IV, V, VI, and VIII) were purchased from Sigma (St. Louis, MO), and solutions were prepared and stored according to the manufacturer’s instructions. Equimolar concentrations (50 nM) of all collagens were dissolved in 100 mM Tris-HCl, pH 9.0, distributed into PolySorp (Nunc, Roskilde, Denmark) microtiter plates, and incubated overnight at 4°C. Wells coated with collagen were blocked for 1 h at room temperature (RT) with 2.5% bovine serum albumin (BSA) dissolved in phosphate-buffered saline (PBS). In parallel, *M. catarrhalis* strains grown overnight were also blocked with PBS-BSA for 1 h at RT. The first row of the microtiter plate was filled with 1 × 10^7^ CFU/ml, and in subsequent rows, serial 3-step dilutions were performed. Plates were incubated for 1 h at RT, followed by washing (4 times) with PBS containing 0.05% Tween 20 (PBST). In the next step, primary rabbit anti-*Moraxella* polyclonal antibodies (PAbs) (5) were added to wells in 100 µl PBS-BSA, followed by incubation for 1 h at RT. Subsequently, plates were washed three times in PBST, followed by the addition of horseradish peroxidase (HRP)-conjugated swine anti-rabbit PAbs (Dako, Glostrup, Denmark) and incubation for 1 h at RT. Finally, plates were washed three times in PBST, developed, and read at 405 nm in a microtiter plate reader (Sunrise Tecan, Männedorf, Germany).

Glass slides were coated with 100 nM collagen or human serum albumin (HSA) as a control and dried at RT. Slides were thereafter washed 3 times in PBS and incubated with *M. catarrhalis* at 10^7^ CFU/ml in PBS in a sterile petri dish. After 1 h of incubation at RT, slides were washed 3 times in PBS and adhered bacteria were fixed by heat in a flame. To visualize bacteria, conventional Gram staining was performed, followed by photography in a light microscope (Olympus IX73; Olympus, Tokyo, Japan).

### Protein-protein interaction assays.

UspA2 (amino acids 30 to 539 [UspA2^30–539^]) and UspA2H (amino acids 50 to 720) full-length proteins were expressed and purified as described previously (5, 6). Recombinant UspA2 and UspA2H were labeled with ^125^I by using the tosylchloramide (chloramine-T) method as described elsewhere (6). PolySorp microtiter plates with separable wells (Nunc-Immuno, Roskilde, Denmark) were coated with collagens (50 nM), and the binding of ^125^I-labeled UspA2 or UspA2H was estimated as described previously (15).

### Fibroblast cultures and adherence of *M. catarrhalis.*

Fibroblasts from lung tissues (23) were cultured in Dulbecco modified Eagle medium (DMEM) (Gibco; Life Technologies, Grand Island, NY) supplemented with glucose and pyruvate in a humid atmosphere with 5% CO_2_ at 37°C. For infection experiments, cells were seeded into 24-well plates and grown until cells covered the plastic surface. Subsequently, 10^7^ CFU/ml of bacteria was added to each well and the plates incubated for 1 to 2 h at 37°C. Wells were washed 5 times with PBS in order to remove unbound bacteria. Trypsin (50 µl) was added to each well, and after dilution, cells were plated onto chocolate agar plates. After incubation at 37°C overnight, CFU were counted manually.

### Confocal microscopy.

Fibroblasts (primary cells) were cultured on glass cover slips. After the cells reached 70 to 80% confluence, coverslips were washed 3 times in PBS and fixed with 4% paraformaldehyde for 10 min. Bacteria were labeled with FM 4-64 (*N*-[3-triethylammoniumpropyl]-4-{6-[4-(diethylamino) phenyl] hexatrienyl} pyridinium dibromide); Life Technologies) and washed 3 times in PBS to remove excess dye. UspA2^30–539^ was labeled with fluorescein isothiocyanate (FITC) according to the manufacturer’s instructions (Sigma). The unincorporated FITC from the UspA2-FITC complex was removed on a D-10 column (Sephadex G-25; GE Healthcare Life Sciences, Freiburg, Germany). To determine adherence, coverslips were blocked with 1% BSA in PBS for 1 h and incubated with 10^7^ CFU/ml FM 4-64-labeled bacteria in 1% BSA for 1 h at RT. Unbound bacteria were removed by washing 3 times in PBS. Thereafter, 5 nM UspA2-FITC solution in 1% BSA was added. After 30 min of incubation, the coverslips were washed 5 times with PBS and mounted in fluorescent mounting medium (Dako) containing 0.3 µg/ml DAPI (4′,6-diamidino-2-phenylindole dihydrochloride). Images were taken in a confocal Zeiss Axio Observer microscope combined with a laser module for LSM 700 (Carl Zeiss, Göttingen, Germany) and processed using Zen Software.

### COPD mouse model.

C57BL/6 female mice (6 to 8 weeks old) were obtained from Jackson Laboratories (Bar Harbor, ME) and housed under standard pathogen-free conditions in an animal facility at Lund University. Mice were kept in 12-h light-dark cycles and fed with standard rodent chow and water *ad libitum*. All experiments were performed according to the animal research ethics guidelines (Canadian Council on Animal Care; www.ccac.ca). For the COPD model, animals were exposed to smoke from 12 3R4F reference cigarettes without filters (Tobacco and Health Research Institute, University of Kentucky, Lexington, KY) in an SIU-48 whole-body cigarette smoking machine (Promech Lab, Vintrie, Sweden). Mice were exposed for 50 min twice daily for 24 weeks. Control mice were exposed to normal room air only. Mice (*n* = 3 per group) were anesthetized to a surgical plane using isoflurane. The tracheas were exposed surgically, and bacteria were injected intratracheally. The wound was closed with 5.0 silk sutures, and the animals were allowed to recover. After 30 min, the animals were sacrificed by CO_2_ exposure, and tracheas, along with lungs, were excised. For estimation of contaminating normal flora, one group of mice was injected with PBS alone and treated similarly as described above. Tracheal tissues were processed for SEM. In parallel, the right lung lobe was excised and homogenized by using a Multi-Gen 7 homogenizer (Pro Scientific, Monroe, CT) in 1 ml chilled PBS, pH 7.4. Lung homogenates (100 µl) were mixed with 100 µl PBS containing 6.86% sucrose. Thereafter, samples were serially diluted and plated on chocolate agar plates. After incubation overnight at 37°C in 5% CO_2_, CFU were determined by manual counting. All experiments were performed in triplicates.

### TEM and SEM.

The interactions of UspA1, UspA2, and UspA2H with collagens were analyzed by negative staining and TEM. Bacterial proteins were labeled with colloidal gold nanoparticles (10 nm; British Biocell International, Cardiff, United Kingdom) as described elsewhere (37). Collagens I, II, IV, and VI were absorbed into 400-mesh carbon-coated copper grids and stained with 0.75% (wt/vol) uranyl formate as recently described in detail (38). The grids were incubated with gold-labeled UspA(s), and after washing, the samples were visualized in a Philips/FEI CM 100 TWIN transmission electron microscope (FEI, Hillsboro, OR) at 60-kV accelerating voltage. Images were recorded with a side-mounted Olympus Veleta camera with a resolution of 2,048 by 2,048 pixels (2K by 2K) using iTEM software. For bacterial interactions, fibroblasts or tissue sections obtained with permission (LU339-00) from the Ethical Committee on Animal Experiments, the Swedish Board of Agriculture, were incubated with 2 × 10^9^ CFU/ml of bacteria for 1 h at 37°C. Unbound bacteria were washed with PBS, and specimens were fixed with 4% formaldehyde and 2.5% glutaraldehyde in PBS for 2 h at RT. Mouse tracheal specimens were fixed overnight in 2.5% glutaraldehyde in cacodylate buffer and washed with cacodylate buffer. Subsequently, samples were dehydrated by using an ascending ethanol series from 50% (vol/vol) up to absolute ethanol and then dried with carbon dioxide. Finally, tissue samples were mounted on aluminum holders, sputtered with 20-nm palladium-gold, and examined in a Philips/FEI XL-30 field emission scanning electron microscope using an Everhart-Tornley secondary electron detector. Images were processed using Scandium software. The electron microscopic work was performed at the Core Facility for Integrated microscopy (Panum Institute, University of Copenhagen). Images were enhanced with pseudocolors in Adobe Photoshop CS6 to visualize bacteria.

### Statistical analysis.

Data were analyzed by using GraphPad Prism 6. The statistical analysis was performed according to the data setup by using one-way or two-way analysis of variance (ANOVA). Evaluation of the EM data was performed by counting bacteria in at least 50 different cellular profiles from three different experiments. Data sets were also evaluated by Student’s *t* test for paired data.

## SUPPLEMENTAL MATERIAL

Figure S1  Adherence of *M. catarrhalis* clinical isolates to various collagens. (A and B) Binding of ^125^I-labeled collagens to *M. catarrhalis* clinical isolates that express UspA2 (A) or UspA2H (B). The data presented in Fig. 1E are plotted here in order to estimate the binding of individual isolates with different collagens. Download Figure S1, TIF file, 0.3 MB

Figure S2  Quantification of gold-labeled UspA1, UspA2, and UspA2H in TEM images shown in Fig. 4C and D and 5C and D. (A) Binding of gold-labeled UspA1, UspA2, and UspA2H to collagens I and II. For quantification, 50 different regions (see Fig. 4C and D) were randomly selected, and gold particles were counted. (B) Quantification of gold particles from TEM images shown in Fig. 5C and D. Error bars indicate standard deviations. Statistical analyses were performed using Student’s *t* test. ***, *P* ≤ 0.001. Download Figure S2, TIF file, 0.1 MB

Figure S3  Adherence of *M. catarrhalis* to primary fibroblasts is dependent on UspA2 and UspA2H, as revealed by confocal microscopy. Fibroblasts were grown on coverslips and incubated with FM 4-64-labeled bacteria (red). After incubation, the ECM, including collagen fibrils, were visualized by FITC-labeled UspA2^30–539^ (green). Orange spots in overlays illustrate colocalization of *M. catarrhalis* and ECM, including collagens. The size bar represents 10 µm. Download Figure S3, TIF file, 1.9 MB
