# Supplementary figures and images for: The Respiratory Pathogen Moraxella catarrhalis Targets Collagen for Maximal Adherence to Host Tissues
Source: mBio. 2016 Mar 22;7(2):e00066-16. doi: 10.1128/mBio.00066-16 (PMC4807357; doi:10.1128/mBio.00066-16)

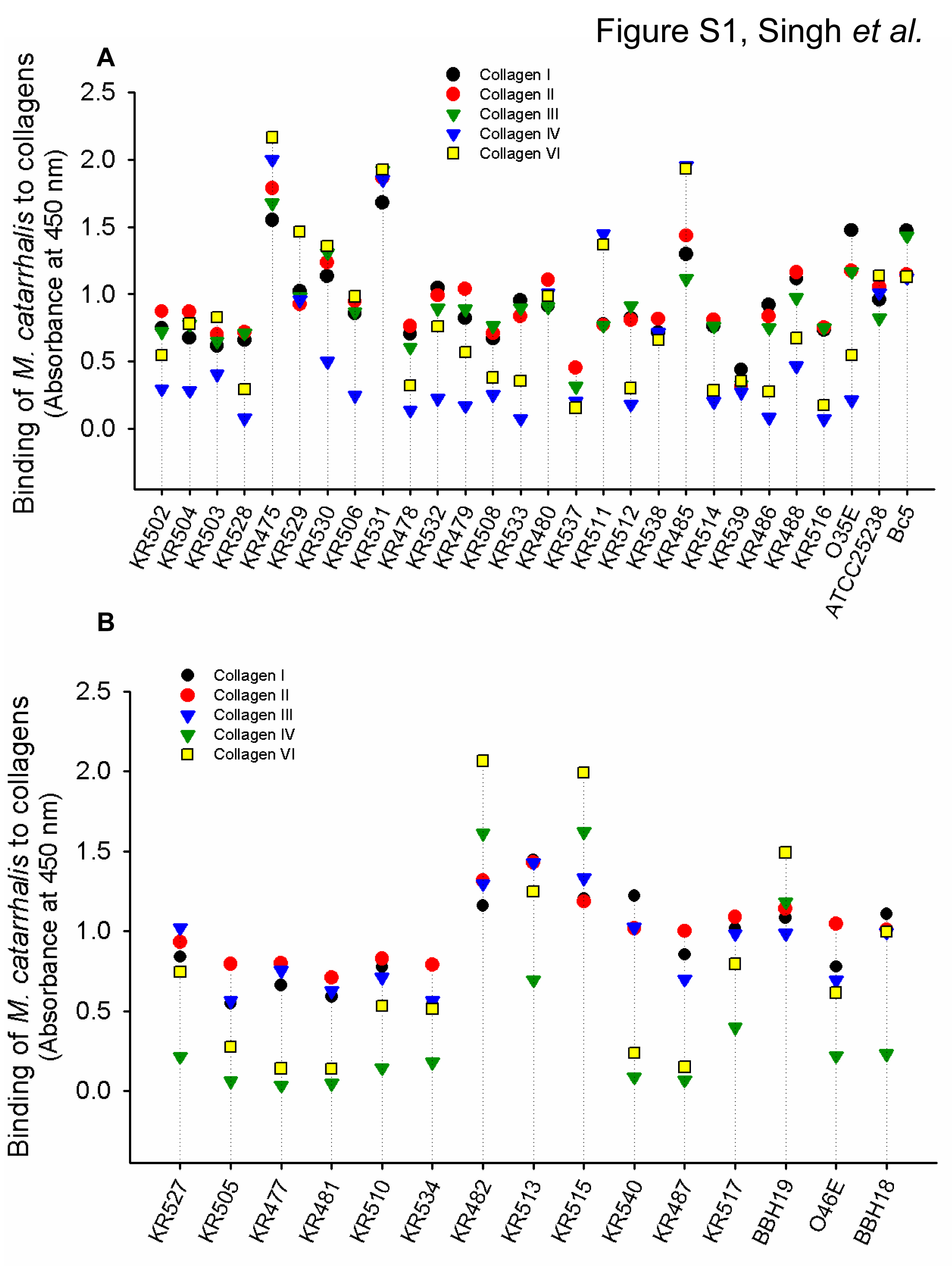

Supplement: Figure S1 — Adherence of M. catarrhalis clinical isolates to various collagens. (A and B) Binding of 125I-labeled collagens to M. catarrhalis clinical isolates that express UspA2 (A) or UspA2H (B). The data presented in Fig. 1E are plotted here in order to estimate the binding of individual isolates with different collagens. Download [file mbo002162739sf1.tif]

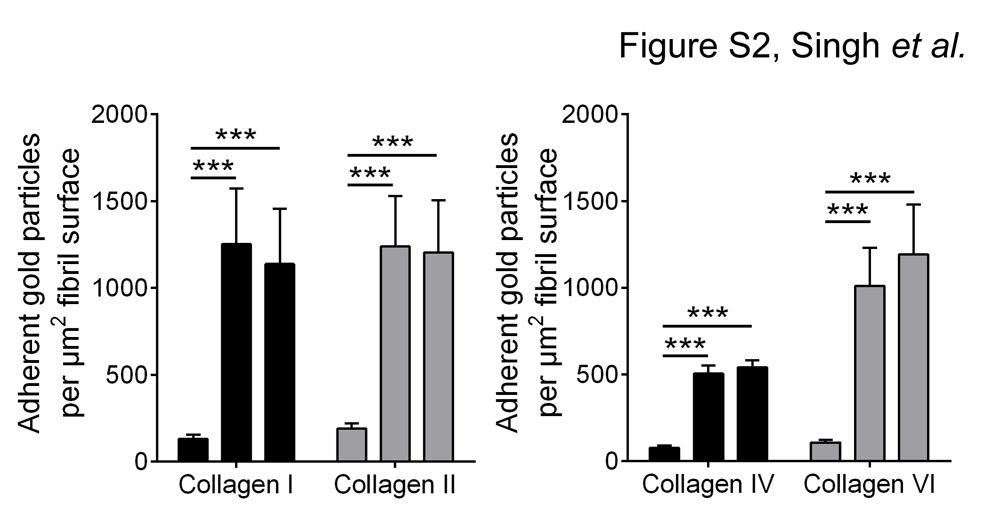

Supplement: Figure S2 — Quantification of gold-labeled UspA1, UspA2, and UspA2H in TEM images shown in Fig. 4C and D and 5C and D. (A) Binding of gold-labeled UspA1, UspA2, and UspA2H to collagens I and II. For quantification, 50 different regions (see Fig. 4C and D) were randomly selected, and gold particles were counted. (B) Quantification of gold particles from TEM images shown in Fig. 5C and D. Error bars indicate standard deviations. Statistical analyses were performed using Student’s t test. ***, P ≤ 0.001. Download [file mbo002162739sf2.tif]

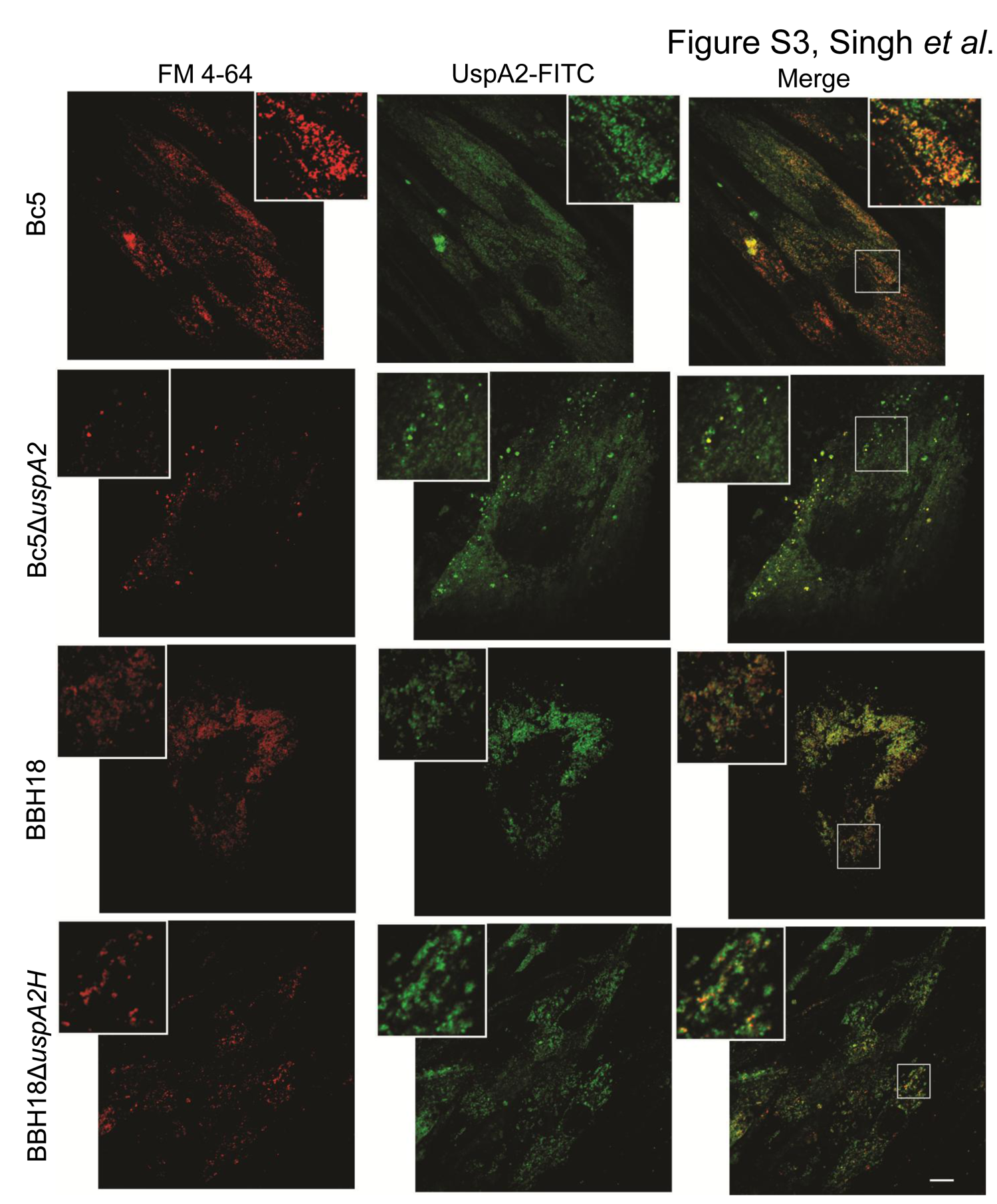

Supplement: Figure S3 — Adherence of M. catarrhalis to primary fibroblasts is dependent on UspA2 and UspA2H, as revealed by confocal microscopy. Fibroblasts were grown on coverslips and incubated with FM 4-64-labeled bacteria (red). After incubation, the ECM, including collagen fibrils, were visualized by FITC-labeled UspA230–539 (green). Orange spots in overlays illustrate colocalization of M. catarrhalis and ECM, including collagens. The size bar represents 10 µm. Download [file mbo002162739sf3.tif]
